# Ploidy Determination in the Pathogenic Fungus *Sporothrix* spp.

**DOI:** 10.3389/fmicb.2019.00284

**Published:** 2019-02-25

**Authors:** Beatriz H. Ferreira, Jorge H. Ramírez-Prado, Gabriela W. P. Neves, Egídio Torrado, Paula Sampaio, Maria Sueli S. Felipe, Ana Tereza Vasconcelos, Gustavo H. Goldman, Agostinho Carvalho, Cristina Cunha, Leila M. Lopes-Bezerra, Fernando Rodrigues

**Affiliations:** ^1^ Life and Health Sciences Research Institute (ICVS), School of Medicine, Braga, Portugal; ^2^ ICVS/3B’s - PT Government Associate Laboratory, University of Minho, Braga, Portugal; ^3^ Biotechnology Unit, Yucatan’s Center for Scientific Research (CICY), Merida, Mexico; ^4^ Laboratory of Cellular Mycology and Proteomics, Biology Institute, University of Rio de Janeiro State, Rio de Janeiro, Brazil; ^5^ Centre of Molecular and Environmental Biology (CBMA), Department of Biology, University of Minho, Braga, Portugal; ^6^ Genomic Science and Biotechnology, Catholic University of Brasília, Brasília, Brazil; ^7^ Department of Cellular Biology, University of Brasília, Brasília, Brazil; ^8^ Laboratório de Bioinformática, Laboratório Nacional de Computação Científica, Petrópolis, Brazil; ^9^ Faculdade de Ciências Farmacêuticas de Ribeirão Preto, Universidade de São Paulo, São Paulo, Brazil; ^10^ Faculdade de Ciências Farmacêuticas, Universidade de São Paulo, São Paulo, Brazil

**Keywords:** sporotrichosis, *Sporothrix schenckii* complex, *S. brasiliensis*, *S. schenckii*, *S. globosa*, *S. pallida*, ploidy, yeast phase

## Abstract

The pathogenic clade of the *Sporothrix* genus comprises the etiological agents of sporotrichosis, a worldwide emergent disease. Despite the growing understanding of their successful pathogen traits, there is little information on genome sizes and ploidy within the genus. Therefore, in this work, we evaluated the ploidy of four species of the *Sporothrix* genus, specifically *Sporothrix brasiliensis*, *Sporothrix schenckii*, *Sporothrix globosa*, and *Sporothrix pallida*. Through cell cycle analysis of the yeast-phase cells, we showed that the DNA content of G_0_/G_1_ cells was similar to the genome size determined by whole genome sequencing. Moreover, ploidy of *S. schenckii*, *S. brasiliensis*, and *S. pallida* that was determined by allele composition using next-generation sequencing (NGS) data is consistent with monomorphic positions at each allele. These data show that the analyzed strains of *Sporothrix* are haploid, or at least aneuploid, thereby laying the foundation for the development of a molecular toolbox for *Sporothrix* spp.

## Introduction

Fungi of the genus *Sporothrix* comprise four species of a pathogenic clade that are the etiological agents of sporotrichosis, a chronic subcutaneous infection that affects humans and other mammals (Teixeira et al., 2014; de Beer et al., 2016). Sporotrichosis is the most prevalent subcutaneous mycosis worldwide, despite being frequently neglected (Queiroz-Telles et al., 2017). Although this mycosis is mainly attributed to *Sporothrix schenckii*, new clinically relevant species are emerging, namely, *Sporothrix globosa*, *Sporothrix lurei*, and *Sporothrix brasiliensis*, the latter being the most virulent species (Marimon et al., 2007; Arrillaga-Moncrieff et al., 2009; Castro et al., 2013; Zhang et al., 2015).

Infection usually results from the direct inoculation of fungal cells into traumatized cutaneous lesions arising *via* plant- or animal-invoked injuries (Rodrigues et al., 2016). Subsequently, in the majority of cases, nodules develop in the infection site leading to ulceration (Kauffman, 1999). Poorly controlled sporotrichosis can disseminate to distant anatomical sites, including bones, lungs, and central nervous system (Ferreira et al., 2016; Hassan et al., 2016; Rodrigues et al., 2016; Mialski et al., 2018). Apart from being a global health problem, sporotrichosis is hyperendemic in Brazil, mainly due to the zoonotic and crossed transmission of *S. brasiliensis* from infected felines (Montenegro et al., 2014; Sanchotene et al., 2015; Gremião et al., 2017). This mycosis is also endemic in Asia, where regions with high incidence of *S. globosa* infection are reported (Zhang et al., 2015). Species of the *Sporothrix* genus exhibit thermal dimorphism, with a yeast phase at 37°C, while at 25°C, the filamentous form is predominant (Kauffman, 1999). Similar to other human pathogens, this dimorphic behavior is an important factor for virulence (Lopes-Bezerra et al., 2006; Téllez et al., 2014). Accordingly, not only yeast cells can be mediators of zoonotic transmission (Gremião et al., 2017), but also mycelium can be present in the environment and transmitted by cat scratches.

The genome structure of *S. schenckii*, *S. brasiliensis*, and *S. globosa* is similar with a total genome length of 32.4, 33.2, and 33.4 Mb, respectively (Teixeira et al., 2014; Huang et al., 2016). On the other hand, *S. pallida* has a genome length of 37.8 Mb, while *S. mexicana* genome size is still unknown (D’Alessandro et al., 2016). Moreover, *S. schenckii* is reported to have high genetic diversity, compared to *S. brasiliensis* and *S. globosa* (Rangel-Gamboa et al., 2016, 2018). Analysis of sequence data from *S. globosa* revealed lack of genetic heterozygosity (Huang et al., 2016). Fungi of the *S. schenckii* complex are highly polymorphic regarding chromosome number and size (Marimon et al., 2007; Sasaki et al., 2014). Additionally, *S. schenckii* is thought to be a diploid organism, whereas the ploidy of other species of the complex has not been described so far (Torres-Guerrero, 1999).

Therefore, in the present work, we developed a flow cytometry (FCM) protocol for cell cycle analysis (Almeida et al., 2007) in order to determine the DNA content per cell (DNA_C_) of *S. brasiliensis*, *S. schenckii*, *S. globosa*, and *S. pallida* yeast cells. Ploidy of the analyzed strains was determined from the comparison of the DNA_C_ with the previously reported genome size, and further validated for *S. schenckii*, *S. brasiliensis*, and *S. pallida* with the analysis of the distribution of base frequencies at variable sites in the genome using the next-generation sequencing (NGS) data.

## Materials and Methods

### Microorganisms and Culture Media

The strains of the *Sporothrix* genus used in this study are listed in Table 1. Yeast cells were maintained at 37°C in yeast extract peptone dextrose (YPD) solid medium (2% glucose, 1% peptone, 0.5% yeast extract, and 1.5% agar; pH = 7.8). For the subsequent assay, yeast cells were cultured in YPD liquid medium at 37°C with aeration on a mechanical shaker (150 rpm). Conidia were obtained after incubation in YPD liquid medium at 25°C with mechanical aeration (150 rpm) for 3 days. Conidia were recovered through successive gaze filtration. *Aspergillus nidulans* A4 and R21/R153 (dos Reis et al., 2018), haploid and diploid strains, respectively, were maintained in YPD solid medium at 30°C. For subsequent analysis, conidia were collected and washed with phosphate buffered saline (PBS) (1×) (8 g NaCl, 0.2 g KCl, 1.44 g Na_2_HPO_4_, 0.24 g KH_2_PO_4_ per liter of sterilized water).

**Table 1:** *Sporothrix* genus strains analyzed during this study.

Isolate identification	Isolation	Source	Reference
***S. brasiliensis***			
ATCC MYA-4823	Brazil	Feline skin lesion	Teixeira et al., 2014
IPEC 27454	Brazil	Feline skin lesion	Almeida-Paes et al., 2014
HUPE 114158	Brazil	Human cutaneous lesion	Castro et al., 2013
IPEC 25374	Brazil	Feline skin lesion	Almeida-Paes et al., 2014
***S. schenckii***			
ATCC MYA-4821	USA	Human, subcutaneous lesion	Teixeira et al., 2014
***S. globosa***			
14879/07	Colombia	Clinical	Oliveira et al., 2015
***S. pallida***			
MUM 17.04	UK	Environment	Kreisel and Schaver, 1985

### Measurements of DNA Content per Cell

The DNA content of isolated cells was determined accordingly to the protocol described in Almeida et al. (2007) with modifications. Exponentially grown yeast cells and conidia were collected, centrifuged (13,000 rpm for 3 min), and washed with PBS (1×). To obtain an uniform single-cell suspension, collected cells were filtered through sterile gauze and fixed overnight with ethanol 70% (vol/vol) at 4°C. Afterwards, cells were harvested, washed, and resuspended in 850 μl of sodium citrate buffer (50 mM; pH = 7.5). Briefly sonicated *Sporothrix* spp. cells were treated at 50°C for 4 h with RNase A (0.50 mg/ml) (GRiSP, Porto, Portugal) and for 2 h with proteinase K (1 mg/ml) (GRiSP). *A. nidulans* conidia, after a brief sonication, were treated at 50°C for 2 h with RNase A (0.50 mg/ml) (GRiSP) and for 2 h with proteinase K (1 mg/ml) (GRiSP). A volume of 50 μl of treated cells was stained overnight with SYBR Green I (10,000×) (Invitrogen, CA, USA) at 4°C. For the yeast cells, a concentration of SYBR Green I of 2% (vol/vol) was used from a 10-fold diluted working solution in Tris-EDTA (pH 8.0) (Sigma-Aldrich). For the case of conidia, a final concentration of 12% (vol/vol) of SYBR Green I (10,000×) was used. Prior to flow cytometry (FCM) analysis, 750 μl of sodium citrate buffer (50 mM; pH 7.5) with 0.25% (vol/vol) of Triton X-100 (Sigma-Aldrich) was added to the stained cells.

### Flow Cytometry

Stained cells were analyzed in a FACS LSRII flow cytometer (Becton Dickinson, NJ, USA) with a 488 nm excitation laser. Signals from a minimum of 30,000 cells per sample were captured in FITC channel (530 nm ± 30 nm) at low flow rate of about 1,000 cells/s and an acquisition protocol was defined to measure forward scatter (FSC) and side scatter (SSC) on a four-decade logarithmic scale and mean green fluorescence intensity of SYBR Green I (MFI_SGI_) on a linear scale. FACS Diva was used as the acquisition software. Results were analyzed with FlowJo (Tree Star, OR, USA) software, version 10, and the coefficients of variation (CV), as well as MFI_SGI_, were estimated using Modfit LT software (Verity Software House, ME, USA).

### Fluorescence Microscopy

Yeast cells of genome strains, *S. schenckii* ATCC MYA-4821 and *S. brasiliensis* ATCC MYA-4823, and *S. pallida* MUM 17.04 labeled with SYBR Green I were analyzed using a fluorescence microscope (DP71 Olympus). The images (1,036 × 1,024 pixels) were acquired with an oil immersion objective (100×/1.40) and analyzed with cellSens imaging software (Olympus Life Science, Tokyo, Japan).

### Ploidy Estimation by Next-Generation Sequencing Data

Raw sequencing data for *S. schenckii* (SRX342487) and *S. pallida* (SRX550176) were downloaded, in FASTQ format, from NCBI’s Sequence Read Archive through the SRA-toolkit (Leinonen et al., 2011). The original NGS sequencing files for *S. brasiliensis* were used (Teixeira et al., 2014). As controls, the raw sequencing data for *Aspergillus flavus* NRRL 3357 (haploid, SRX2124714) and *Candida albicans* SC 5314 (diploid, SRX2250255) were also obtained from NCBI-SRA.

Short raw reads for each species were aligned to the corresponding assembled genome (*S. schenckii* 1,099–18 GCF_000961545.1, *S. brasiliensis* 5110 GCA_000820605.1, *S. pallida* SPA8 GCA_000710705.2, *A. flavus* NRRL357 GCF_000006275.2, and *C. albicans* SC5314 GCF_000182965.3) with Bowtie2 (Leinonen et al., 2011) using the “very-sensitive” option. If raw reads were paired, “no-mixed, no-discordant, no-overlap, no-contain” parameters were also used. For each genome, the resulting SAM files were converted to BAM files and sorted with samtools (Li et al., 2009), and then split by chromosomes/scaffolds using bamtools (Barnett et al., 2011).

Allele frequencies at every position along each chromosome/scaffold for all genomes were calculated using the “ploidyNGS” algorithm (Corrêa dos Santos et al., 2017), with default parameters (max_allele_freq 0.95, max_depth 100). Chromosome/scaffold frequency files for each genome were then merged. Ploidy was estimated by constructing histograms from the frequency files and comparing to the haploid and diploid controls.

### Statistical Analysis

Data are reported as the mean ± standard deviation (SD) of at least three independent assays. Data were analyzed using MS Excel 2016 (Office, Microsoft^®^, WA, USA) and GraphPad Prism Software, version 7.0 (GraphPad Software Inc., CA, USA). A linear regression was calculated to predict the MFI_SGI_ based on genome size (Mb).

## Results and Discussion

### The DNA Content of *Sporothrix* spp. Yeast Cells

**Figure 1:**
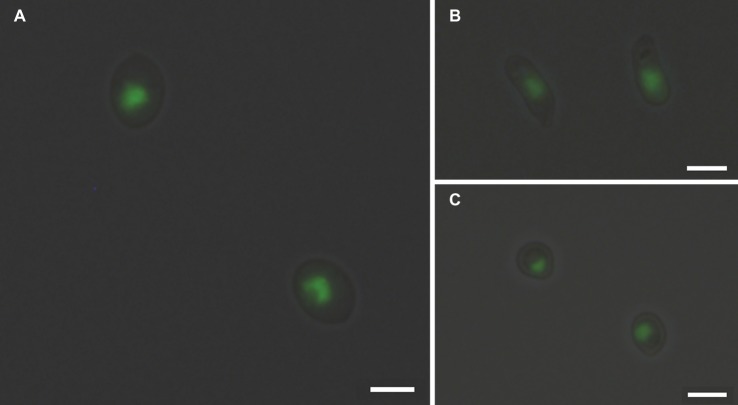
SYBR Green I nuclear staining of *Sporothrix* spp. Fluorescence microscopy analysis of **(A)** *S. brasiliensis* ATCC MYA-4823, **(B)** *S. schenckii* ATCC MYA-4821, and **(C)** *S. pallida* MUM 17.04 stained with SYBR Green I reveals the presence of mononucleated yeast cells. Images (1,036 × 1,024 pixels) were acquired in a fluorescence microscope (DP71 Olympus) with an oil immersion objective (100×/1.4) and cropped using the cellSens imaging software (Olympus Life Science). Scale bar equals 3 μm.

Measuring the DNA content is a well-established method to monitor cell proliferation, cell cycle, and cell ploidy. Despite the relevance of *Sporothrix* spp. in human and animal diseases, ploidy determination in species of this genus is scarce. Indeed, the diploid status of *S. schenckii* has been reported (Torres-Guerrero, 1999). To determine the DNA content of the yeast cells of different species of the *Sporothrix* genus, a protocol for cell cycle analysis using FCM (Fortuna et al., 2000; Almeida et al., 2007) was applied with modifications to obtain lower coefficients of variation. The CV is a measurement of the peak width of DNA from cells in G_0_/G_1_ phase of the cell cycle and therefore, low CV values are critical for the accuracy of cellular ploidy determination (Fortuna et al., 2000; Almeida et al., 2007). A minimum of cellular debris and clumps was achieved by using exponentially growing cells and gauze filtration (data not shown). Cell separation and the absence of filamentous forms were confirmed by optical microscopy. The RNase A and proteinase K treatments were optimized to achieve CVs lower than 7, as described elsewhere (Table 2; Rodrigues et al., 2003; Almeida et al., 2007). Additionally, different SYBR Green I concentrations – 0.2, 2, 4, and 10% – were used for DNA staining, ensuring that the dye was not limiting (data not shown). Fluorescence microscopy analysis revealed specific nuclear staining, as expected for SYBR Green I, for the analyzed strains (Figure 1).

**Table 2:** Mean fluorescent intensity and corresponding calculated DNA content per cell (DNA_C_) (Mb) of *Sporothrix* genus strains estimated by flow cytometry (FCM) of SYBR Green I stained cells.

Isolate identification	(MFI_SGI_ ± SD^a^) × 10^3^	CV^b^	DNA_C_ ± SD (Mb)^c^	Genome size (Mb)^d^	Ploidy ratio^e^
***S. brasiliensis***					
ATCC MYA-4823	101.13 ± 0.58	4.42	29.89 ± 0.10	33.2	0.9
IPEC 27454	89.26 ± 1.09	3.49	25.43 ± 0.11	–	0.8
HUPE 114158	88.97 ± 0.13	4.00	25.32 ± 0.11	–	0.8
IPEC 25374	100.27 ± 0.36	4.28	29.57 ± 0.10	–	0.9
***S. schenckii s. str.***					
ATCC MYA-4821	85.80 ± 0.84	3.53	24.13 ± 0.12	32.4	0.7
***S. globosa***					
CBS 120340	N.D.	N.D.	N.D.	33.4	N.D.
14,879/07	97.15 ± 0.33	5.65	28.40 ± 0.10	–	0.9
***S. pallida***					
SPA8	N.D.	N.D.	N.D.	37.8	N.D.
MUM 17.04	109.71 ± 3.28	6.40	33.12 ± 0.10	N.D.	0.9

a Mean fluorescence intensity (MFI_SGI_) of cells in G_0_/G_1_ phases of the cell cycle profile.

b Coefficient of variation (CV) for the G_0_/G_1_ cell population.

c DNA content per cell, in megabases, estimated by FCM.

d Genome size, in megabases, reported in the literature (Teixeira et al., 2014; D’Alessandro et al., 2016; Huang et al., 2016).

e Ratio of the genome size estimated by FCM and the reported value; N.D., not determined.

**Figure 2:**
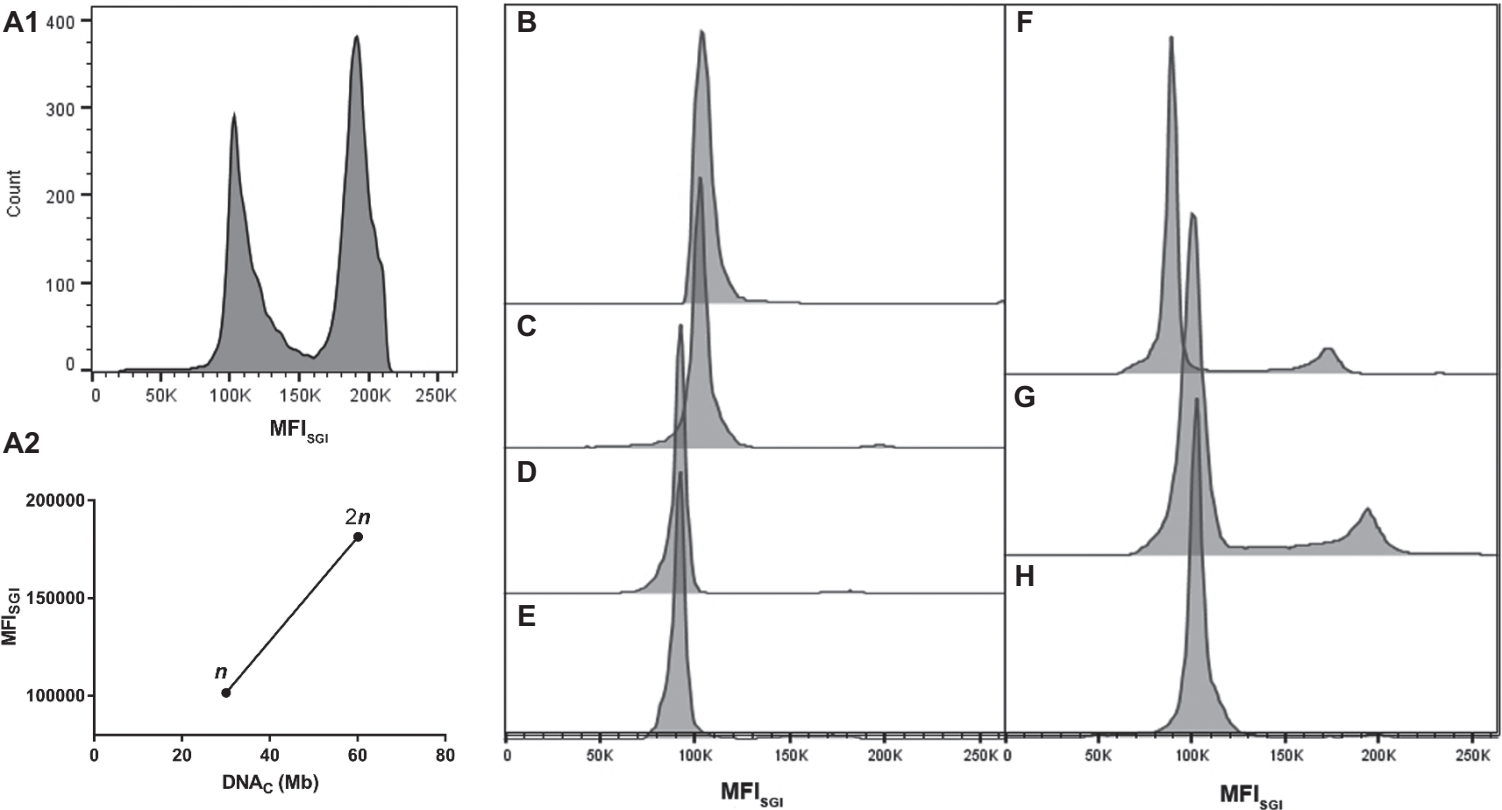
Representative histograms of cell cycle analysis of the analyzed microorganisms. **(A)** Analyses of *A. nidulans* strains: **(A1)** mixed populations of *A. nidulans* haploid and diploid strains and **(A2)** graph showing a typical standard curve relating MFI_SGI_ of *n* and 2*n* peaks of *A. nidulans* strains and the theoretical amount of DNA per cell (DNA_C_). Histograms for: *S. brasiliensis* **(B)** ATCC MYA-4823, **(C)** IPEC 25374, **(D)** IPEC 27454, and **(E)** HUPE 114158; **(F)** *S. schenckii* ATCC MYA-4821; **(G)** *S. globosa* 14879/07; and **(H)** *S. pallida* MUM 17.04.

FCM allows the establishment of a direct correlation between MFI_SGI_ and the DNA_C_ (Rodrigues et al., 2003). As a first approach, the genome size of *Sporothrix* spp. was estimated using a correlation based in the MFI_SGI_ and the DNA_C_ of haploid and diploid strains of *Saccharomyces cerevisiae* (data not shown). The utilization of this well-established DNA content cell standard would allow the analysis of cell populations with *n*, 2*n*, and 4*n* of DNA content. However, SYBR Green I exhibits a preferential binding to GC-rich sequences (Gudnason et al., 2007), with *S. cerevisiae* and *Sporothrix* spp. presenting different GC content. In fact, whereas for *S. cerevisiae*, the percentage of GC content is around 38%, for *S. brasiliensis* and *S. schenckii*, the values are of 62%, being for *S. globosa* and *S. pallida* of 54 and 52%, respectively (Teixeira et al., 2014; D’Alessandro et al., 2016; Huang et al., 2016). Thus, for the analysis of the cell DNA content of *Sporothrix* spp., we took advantage of haploid and diploid strains of *A. nidulans* as reference, which presents a GC content of 50% (Galagan et al., 2005). As such, from the described haploid genome size of *A. nidulans* (30.07 Mb) (Galagan et al., 2005) and the MFI_SGI_ obtained for each cellular DNA content (*n* and 2*n*, corresponding to G_0_/G_1_ phase of the cell cycle of haploid and diploid *A. nidulans* conidia) (Figure 2A1), a linear regression was established – MFI_SGI_ = (2,659 ± 141.6)*DNA_C_ + (21,647 ± 6,726) (Figure 2A2; *R* ^2^ = 0.9888).

**Figure 3:**
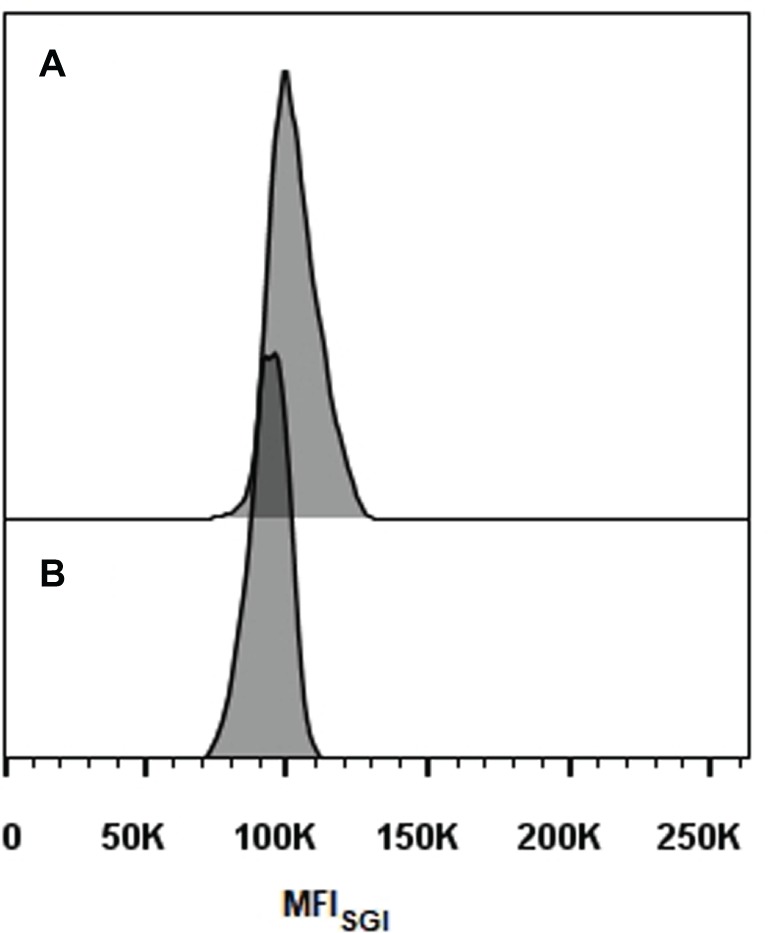
Histograms of cell cycle analysis of *Sporothrix* spp. conidia. **(A)** *S. brasiliensis* ATCC MYA-4823 and **(B)** *S. schenckii* ATCC MYA-4821.

Representative histograms for *S. brasiliensis*, *S. schenckii*, *S. globosa*, and *S. pallida* obtained by FCM are shown in Figures 2B–H, respectively. This figure includes the histograms obtained for the fully sequenced strains: *S. brasiliensis* ATCC MYA-4823 and *S. schenckii* ATCC MYA-4821 (Figures 2B,F, respectively) (Teixeira et al., 2014). The average of the DNA_C_ estimated according to the cells in G_0_/G_1_ phase of the cell cycle for the different strains of *S. brasiliensis* analyzed ranged between 25.32 and 29.89 Mb, and was of 24.13, 28.40, and 33.12 Mb for *S. schenckii*, *S. globosa*, and *S. pallida,* respectively (Table 2). Moreover, *S. brasiliensis* ATCC MYA-4823 and *S. schenckii* ATCC MYA-4821 conidia presented identical values of MFI_SGI_ to those presented for the yeast form (Figure 3). Overall, these data suggest that there are no variations in ploidy in the morphological transition from conidia to yeast cells of these *Sporothrix* spp.

### Ploidy Estimation of *Sporothrix* spp. by Flow Cytometry and Next-Generation Sequencing Data

To determine the ploidy of the analyzed organisms, a comparison was performed between the DNA_C_ of cells in G_0_/G_1_ phase of the cell cycle, determined by FCM (Table 2), and the genome length reported for each organism from the full sequence analysis. The ploidy state of the studied organisms was inferred by the ratio obtained from these two parameters (designed as ploidy ratio) (Almeida et al., 2007). *S. schenckii* presented a ploidy ratio of 0.7, suggestive of a haploid profile, although aneuploidy cannot be excluded (Table 2). In what regards *S. brasiliensis*, the ploidy ratio ranged from 0.9, for the sequenced strain, to 0.8 in other isolates (Table 2). These data point for a haploid profile in these strains. For the analyzed strains of *S. globosa* and *S. pallida*, a ploidy ratio of 0.9 was obtained, suggestive also of a haploid profile. Additionally, a DNA_C_ for *S. mexicana* MUM 17.07 identical to the other isolates tested was obtained suggesting a haploid status, considering a genome length similar to the other *Sporothrix* spp. (data not shown).

**Figure 4:**
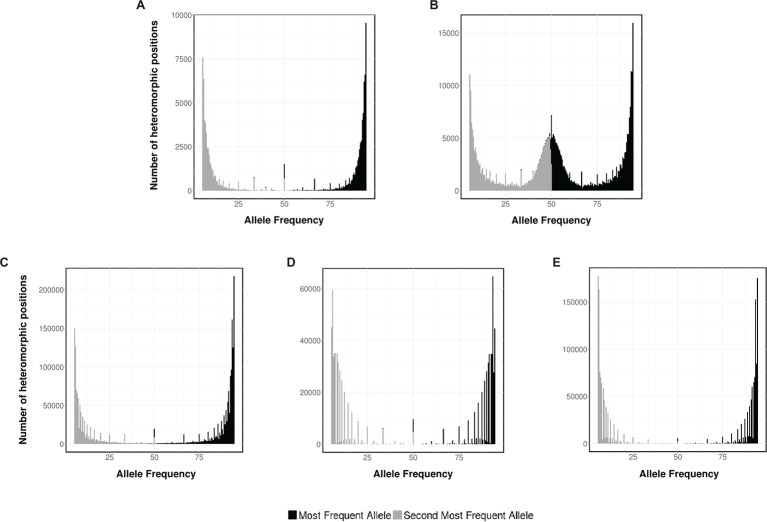
Ploidy estimation by NGS data. Distribution of the two most frequent putative alleles for **(A)** *A. flavus* NRRL 3357, **(B)** *C. albicans* SC 5314, **(C)** *S. schenckii* ATCC MYA-4821, **(D)** *S. brasiliensis* ATCC MYA-4823, and **(E)** *S. pallida* CBS 120340.

Ploidy can be determined indirectly – without measuring cellular DNA_C_ – from the analysis of the short-read sequencing data generated by NGS experiments. Therefore, to strengthen our data, this methodology was also applied to determine the ploidy of *Sporothrix* spp. Briefly, a typical NGS run produce millions of reads coming from every piece of DNA present on the original sample. After mapping, the NGS reads to an assembled genome, the frequency of variations at every position along the genome can be interpreted to be supporting different alleles at each position and used to infer the ploidy level. In the case of a haploid organism, a single allele for the vast majority of reads it is expected (monomorphic positions), whereas for diploids, polymorphic positions are expected where half of the readings support one allele and the other half support an alternative allele. As controls, we analyzed the allele frequency of NGS data from haploid and diploid fungus, *A. flavus* and *C. albicans*, respectively (Figures 4A,B).

As expected, for *A. flavus* the most abundant allele is close to 95% (monomorphic positions). Conversely, for *C. albicans*, the histogram displays monomorphic positions, with a frequency close to 95%, and heterozygous positions, with frequencies close to 50% for the first and second more frequent base. The allele frequency plots for *S. schenckii*, *S. brasiliensis*, and *S. pallida* (Figures 4C–E, respectively) present a single peak for both the most abundant allele (close to 95%) and the second most abundant one (close to 5%), supporting the hypothesis that these three strains of *Sporothrix* spp. are haploid organisms. For *S. globosa*, a low heterozygosity was also previously described (Huang et al., 2016). The differences on the densities of the histograms can be accounted due to the difference on depth coverage and NGS sequencing technology used in each case (*S. schenckii* Illumina HiSeq 2000, *S. brasiliensis* 454 GS FLX, and *S. pallida* Ion Torrent PGM).

## Conclusion

Our results exclude a diploid DNA content for these organisms and propose a haploid or at least a near haploid profile for *S. schenckii*, *S. brasiliensis*, *S. globosa*, and *S. pallida*.

The construction of whole-genome knockout collections (e.g., by random insertion) are invaluable tools for connecting gene sequence to function. The application of such methodologies to identify and characterize virulence traits from *Sporothrix* spp. has not been received significant experimental attention, most likely because *S. schenckii* was thought to be diploid (Torres-Guerrero, 1999). The novel information presented herein represents a timely and practical advance that may now be exploited using molecular techniques like *Agrobacterium*-based transformation methods.

Apart from the biological significance of the ploidy state in the vegetative growth and sexual cycle, this knowledge, coupled to the power of modern molecular technologies, as CRISPR-mediated gene disruption, may open new avenues for the identification of virulence traits of these pathogens.

## Data Availability

The datasets analyzed for this study can be found in the NCBI-SRA (https://www.ncbi.nlm.nih.gov/sra/?term=SRX342487+OR+SRX550176+OR+SRX2124714+OR+SRX2250255) with the following accession codes: SRX342487 for *Sporothrix schenckii*, SRX550176 for *S. pallida*, SRX2124714 for *Aspergillus flavus* NRRL 3357, and SRX2250255 for *Candida albicans* SC 5314. Data for *S. brasiliensis* are available upon request from MF.

## Author Contributions

LL-B and FR conceived the study. BF, JR-P, and GN performed the experiments. BF, JR-P, and FR analyzed the data. BF, JR-P, and ET drafted the manuscript. GG, AC, CC, PS, MF, and AV contributed reagents, materials, and analysis tools. All the authors reviewed the manuscript critically.

### Conflict of Interest Statement

The authors declare that the research was conducted in the absence of any commercial or financial relationships that could be construed as a potential conflict of interest.
